# Nilotinib Enhances the Efficacy of Conventional Chemotherapeutic Drugs in CD34^+^CD38^−^ Stem Cells and ABC Transporter Overexpressing Leukemia Cells

**DOI:** 10.3390/molecules19033356

**Published:** 2014-03-19

**Authors:** Fang Wang, Xiao-Kun Wang, Cheng-Jun Shi, Hui Zhang, Ya-Peng Hu, Yi-Fan Chen, Li-Wu Fu

**Affiliations:** State Key Laboratory of Oncology in Southern China, Cancer Center, Sun Yat-sen University, Guangzhou 510060, China

**Keywords:** nilotinib, leukemia stem cells, multidrug resistance, ATP-binding cassette transporters, ABCG2, ABCB1

## Abstract

Incomplete chemotherapeutic eradication of leukemic CD34^+^CD38^−^ stem cells is likely to result in disease relapse. The purpose of this study was to evaluate the effect of nilotinib on eradicating leukemia stem cells and enhancing the efficacy of chemotherapeutic agents. Our results showed that ABCB1 and ABCG2 were preferentially expressed in leukemic CD34^+^CD38^−^ cells. Nilotinib significantly enhanced the cytotoxicity of doxorubicin and mitoxantrone in CD34^+^CD38^−^ cells and led to increased apoptosis. Moreover, nilotinib strongly reversed multidrug resistance and increased the intracellular accumulation of rhodamine 123 in primary leukemic blasts overexpressing ABCB1 and/or ABCG2. Studies with ABC transporter-overexpressing carcinoma cell models confirmed that nilotinib effectively reversed ABCB1- and ABCG2-mediated drug resistance, while showed no significant reversal effect on ABCC1- and ABCC4-mediated drug resistance. Results from cytotoxicity assays showed that CD34^+^CD38^−^ cells exhibited moderate resistance (2.41-fold) to nilotinib, compared with parental K562 cells. Furthermore, nilotinib was less effective in blocking the phosphorylation of Bcr-Abl and CrkL (a substrate of Bcr-Abl kinase) in CD34^+^CD38^−^ cells. Taken together, these data suggest that nilotinib particularly targets CD34^+^CD38^−^ stem cells and MDR leukemia cells, and effectively enhances the efficacy of chemotherapeutic drugs by blocking the efflux function of ABC transporters.

## 1. Introduction

Intrinsic or treatment-induced acquired resistance is the major reason for therapeutic failure and an important cause of death in acute leukemia patients. One of the best characterized resistance mechanisms is mediated by ATP-binding cassette (ABC) transporters which are capable of recognizing and extruding a broad range of structurally and functionally unrelated compounds [1,2,3,4]. An increasing number of ABC transporters have been shown to cause multidrug resistance (MDR) in leukemia patients. In most of the studies investigating *de novo* or secondary adult acute myeloid leukemia (AML), ABCB1 (ATP-binding cassette superfamily member B1, P-glycoprotein) is an independent prognostic factor associated with reduced remission rates, and in some reports, inferior leukemia-free and overall survival [5,6,7]. Overexpression of ABCB1, ABCC1 (multidrug resistance-associated protein 1, MRP1), ABCC3 (MRP3), and ABCG2 (breast cancer resistance protein, BCRP) genes is associated with poor prognosis in AML patients [8,9,10,11]. High expression of MRP genes is associated with a reduced relapse-free survival in acute lymphoblastic leukemia (ALL) patients and relapsed patients showed a higher expression of MRP genes [12]. ABCB1 expression in *de novo* adult ALL patients is an independent predictor of complete remission achievement [13]. 

A fascinating fact regarding ABC transporters is the documented hyper-expression of some proteins of this family by stem cells. Many types of cancers, including acute leukemia, are organized hierarchically and their growth is sustained by a subpopulation of rare cancer stem cells (or cancer initiating cells) displaying asymmetric cell division, self-renewal capacity, and thus maintenance of disease [14,15]. The existence of cancer stem cells (CSC) was first demonstrated in AML using xenogeneic transplant models. Specifically, the CD34^+^CD38^−^ cells differentiated into leukemic blasts in the recipient mice, and recapitulated the disease observed in the patient. These leukemia stem cells (LSCs) are responsible for the occurrence of metastases and relapses after induction chemotherapy and exhibit intrinsic resistance to treatment [16,17,18,19]. The first property of this population was characterized by their ability to export Hoescht 33342 and rhodamine 123 fluorescent dyes from cells, which are transported by proteins of the ABC superfamily [20]. Accumulating data suggest that ABCB1, and especially ABCG2 are abundantly expressed in the so-called LSCs [21,22,23,24]. De Grouw *et al*. [25] have found higher mRNA levels of 27 ABC transporters in the CD34^+^CD38^−^ cells compared to the more committed CD34^+^CD38^−^ progenitors from untreated AML patients. Additionally, cells that hyper-express ABCB1 show the phenotype CD34^+^ in human hematopoietic stem cells. Though the role of these transporters remains unclear, it is most often assumed that these transport proteins play an important role in protecting stem cells against toxic substances [20,26]. It has also been reported that ABC transporters may be involved in the regulation of key processes of stem cells, perhaps involved in their capacity for self-renewal and differentiation [27]. 

The development of ABC efflux transporter inhibitors has been extensively investigated since 1981 [28]. Till now, three generations of inhibitors have been identified and some of them are currently under clinical trials for specific forms of advanced cancers [29]. Recently, it has been collectively reported that tyrosine kinase inhibitors (TKIs) can overcome ABC-transporters-mediated multidrug resistance by inhibiting their transport activities. Lapatinib has been shown to inhibit the activity of ABCB1, ABCG2 and MRP7 [30,31]. Kitazaki *et al*. [32] and Nakamura *et al*. [33] have reported gefitinib to be an inhibitor of ABCB1 and ABCG2. Other TKIs have also been found to overcome MDR mediated by ABC transporters, including erlotinib, cediranib, vandetanib, sunitinib and so on [34,35,36,37]. We have previously shown that apatinib and axitinib could target side population (SP) phenotype cells and enhance the efficacy of chemotherapeutic drugs [38,39]. 

Nilotinib (AMN107, Tasigna^®^, Novartis), a selective inhibitor of the tyrosine kinase activities of Bcr-Abl, platelet-derived growth factor receptor (PDGFR) and mast/stem-cell growth factor receptor (c-KIT), is an encouraging therapeutic option for chronic myeloid leukemia (CML) patients with imatinib resistance or intolerance. The goal of the current study is to investigate the effect of nilotinib on drug retention in leukemia-initiating CD34^+^CD38^−^ stem cells and characterize the interactions of nilotinib with ABC transporters in primary leukemic blasts.

## 2. Results and Discussion

### 2.1. Nilotinib Enhanced the Efficacy of Chemotherapeutic Drugs in CD34^+^CD38^−^ Cells

**Figure 1:**
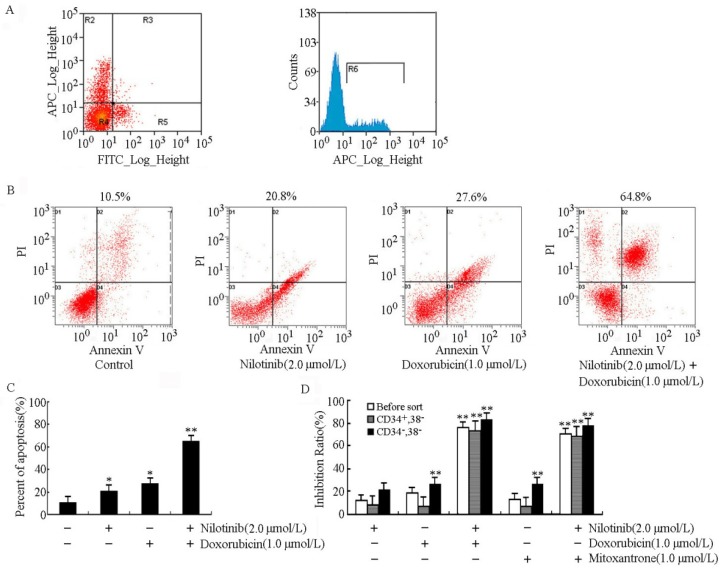
Nilotinib targeted CD34^+^CD38^−^ cells and enhanced the efficacy of doxorubicin and mitoxantrone in the inhibition of proliferation and induction of apoptosis. (**A**) BM mononuclear cells isolated from acute myeloid leukemia patients were co-stained with anti-CD34 and -CD38 antibodies and CD34^+^CD38^−^ cell-enriched subpopulation was further sorted by FACS. (**B**) and (**C**) Sorted CD34^+^CD38^−^ cells treated with doxorubicin and nilotinib in the indicated concentrations for 48 h, respectively. Apoptosis was analyzed by flow cytometry as the percentage of cells labeled by Annexin V and propidium iodide. (**D**) Induction of 50% cell death by doxorubicin and mitoxantrone in the presence of nilotinib at indicated concentrations. Growth inhibition was determined by the MTT assay according to the protocol described in the Experimental section. All these experiments were repeated at least thrice, and a representative experiment is shown. Columns, means of triplicate determinations; * *p* < 0.05; ** *p* < 0.01.

BM mononuclear cells isolated from acute myeloid leukemia patients were co-stained with anti-CD34 and -CD38 antibodies and CD34^+^CD38^−^ hematopoietic cells were flow-cytometrically defined as shown in Figure 1A. In the investigated CD34^+^CD38^−^ cell populations that contained the majority of stem cell activity, only a small percentage of cells underwent apoptosis upon administration of low-dose single-agent nilotinib (20.8% ± 2.9%) and doxorubicin (27.6% ± 3.3%) could be observed. It is interesting to note that nilotinib drastically enhanced the apoptosis of CD34^+^CD38^−^ cells (64.8% ± 3.8%) when used in combination with doxorubicin (Figure 1B,C). As shown in Figure 1D, CD34^+^CD38^−^ cells exhibited higher resistance to doxorubicin and mitoxantrone than CD34^−^CD38^−^ cells. The inhibition ratios of doxorubicin and mitoxantrone at 1.0 μmol/L in CD34^+^CD38^−^ cells were 26.8% ± 3.1% and 26.7% ± 4.8%, while only a slight cytotoxic effect was observed in CD34^+^CD38^−^ cells (the inhibition ratio was 6.9% ± 2.1% and 7.3% ± 1.1% for doxorubicin and mitoxantrone, respectively). Compared to the corresponding monotherapies, nilotinib significantly enhanced the sensitivity of different cell subgroups to doxorubicin and mitoxantrone. In the presence of 2.0 µmol/L nilotinib, the inhibition ratio of doxorubicin and mitoxantrone increased (*a*) from 18.8% ± 3.2% to 75.6% ± 5.1% and from 12.9% ± 2.8% to 70.9% ± 3.6% in un-sorting BM cells; (*b*) from 6.9% ± 2.1% to 73.6% ± 4.9% and from 7.3% ± 1.1% to 68.3% ± 2.2% in CD34^+^CD38^−^ cells; (*c*) from 26.8% ± 3.1% to 82.6% ± 4.5% and from 26.7% ± 4.8% to 77.5% ± 5.0% in CD34^−^CD38^−^ cells (Figure 1D). Together, these data suggested that nilotinib effectively targeted CD34^+^CD38^−^ cells and enhanced the chemosensitivity of antineoplastic drugs. 

### 2.2. Expression Profiles of ABC Transporter Genes in CD34^+^CD38^−^ Cells and Acute Leukemia Patients

**Figure 2:**
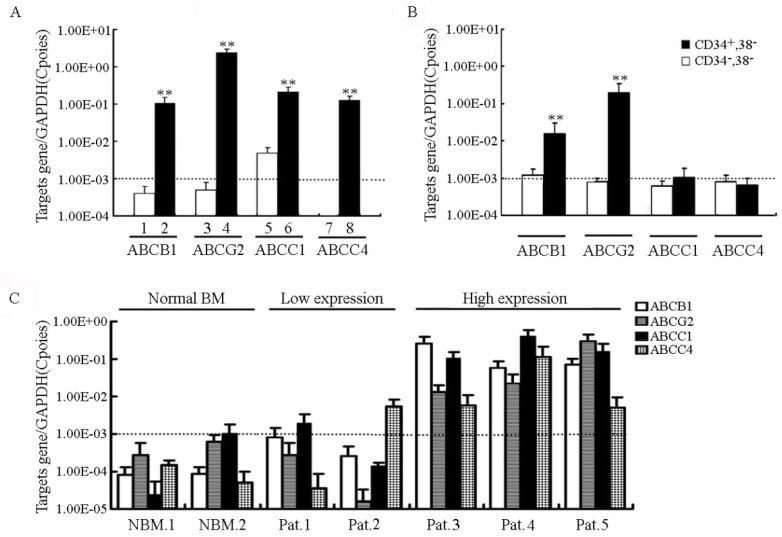
ABC transporters were highly expressed in CD34^+^CD38^−^ cells and *de novo* primary leukemic blasts. (**A**) Detection of ABCB1/P-gp, ABCG2/BCRP, ABCC1/MRP1 and ABCC4/MRP4 expression in ABC transporter overexpressing cells and their parental sensitive cells by quantitative real-time PCR (1, KB; 2, KBv200; 3, S1; 4, S1-M1-80; 5, HL60; 6, HL60/ADR; 7, NIH3T3; 8, NIH3T3/MRP4-2). (**B**) Detection of ABCB1/P-gp, ABCG2/BCRP, ABCC1/MRP1 and ABCC4/MRP4 expression in different hematopoietic cell populations isolated from K562 cells. (**C**) Endogenous expression of ABC transporters in the representative primary leukemic blasts and normal bone marrow samples (NBM, normal bone marrow; Pat., patient). ** *p* < 0.01.

To determine the relationship between stem cells and the MDR phenotype, the gene expression of ABC transporters was assessed in sorted K562 cell subpopulations. KBv200, S1-M1-80, HL60/ADR and NIH3T3/MRP4 cell lines are drug resistant models with overexpression of ABCB1, ABCG2, ABCC1 and ABCC4, respectively. The basal expression of the four transporters in the parental cell lines was nearly undetectable (below 1 × 10^−3^ copies) (Figure 2A). As shown in Figure 2B, the expression of ABCB1 and ABCG2 were significantly higher in CD34^+^CD38^−^ cells compared with more matured CD34^−^CD38^−^ subpopulations. In addition, the expression levels of the four transporters in five *de novo* acute leukemia patients (three of them were diagnosed with AML and two were ALL) and two normal bone marrow (NBM) samples were also detected. All four genes showed higher expression levels in three patients (Pat.3–5) compared to the NBM samples (Figure 2C). These results confirmed that both primitive hematopoietic stem cells and new diagnosed acute leukemia patients showed high expression levels of ABC transporters.

### 2.3. Nilotinib Sensitized the Primary Leukemic Blasts with ABCB1- and ABCG2-Overexpressing to Substrate Anticancer Drugs

**Figure 3:**
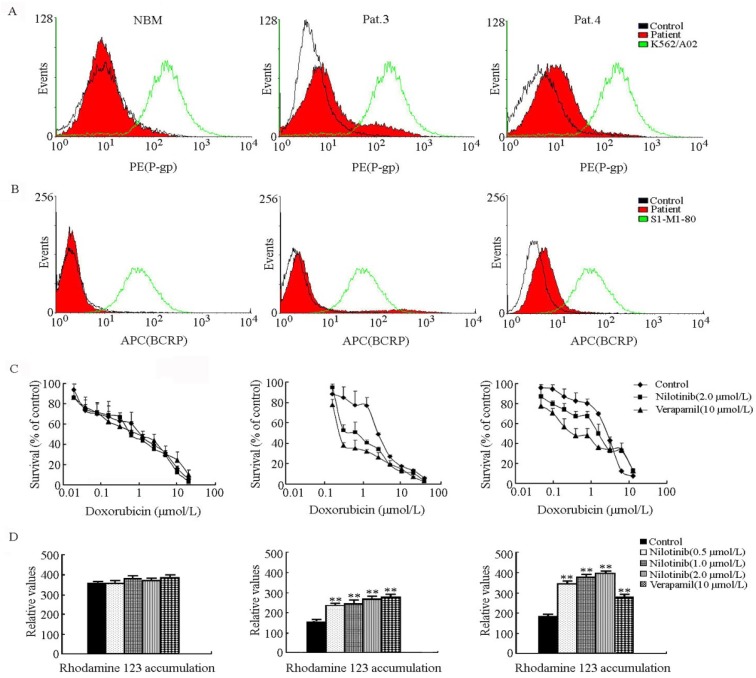
Nilotinib enhanced the cytotoxicity of doxorubicin and the intracellular accumulation of Rho 123 in primary leukemic blasts with ABCB1 and ABCG2 overexpression. Mononuclear cells were isolated as described in the Experimental section. (**A**) Cell surface expression of ABCB1 in representative primary leukemic blasts and normal bone marrow samples was determined by flow cytometry. (**B**) Cell surface expression of ABCG2 in representative primary leukemic blasts and normal bone marrow samples. (**C**) Enhancement of doxorubicin cytotoxicity in primary leukemic blasts by nilotinib. Cytotoxicity was determined by MTT assay as described in the Experimental section. Data represent the mean±standard deviations (SDs) from at least three independent experiments performed in triplicate. (**D**) Intracellular accumulation of Rho 123 was not significantly affected by nilotinib in NBM cells and was increased in a dose-dependent manner in leukemic blasts with ABCB1 and ABCG2 overexpression. ** *p* < 0.01.

The cell surface expression of ABCB1 and ABCG2 was confirmed by flow cytometric analysis in patient 3 (Pat.3) and patient 4 (Pat.4) (Figure 3A,B). As shown in Figure 3C, the IC_50_ values of doxorubicin for normal bone marrow (NBM) blasts, Pat.3 and Pat.4 were 0.948 ± 0.221, 1.329 ± 0.128 and 2.426 ± 0.346 µmol/L, respectively. Nilotinib at 2.0 µmol/L significantly sensitized the MDR cells to doxorubicin treatment as compared to the NBM blasts and the fold-reversals were 2.11 and 6.17 in Pat.3 and Pat.4. The effect of nilotinib on intracellular accumulation of Rho 123 in ABCB1- and ABCG2-overexpressing primary leukemic blasts was also detected. Our results showed that nilotinib enhanced the intracellular accumulation of Rho 123 in a dose-dependent manner (Figure 3D). 

### 2.4. Nilotinib Particularly Reversed ABCB1 and ABCG2-Mediated MDR in Vitro

**Figure 4:**
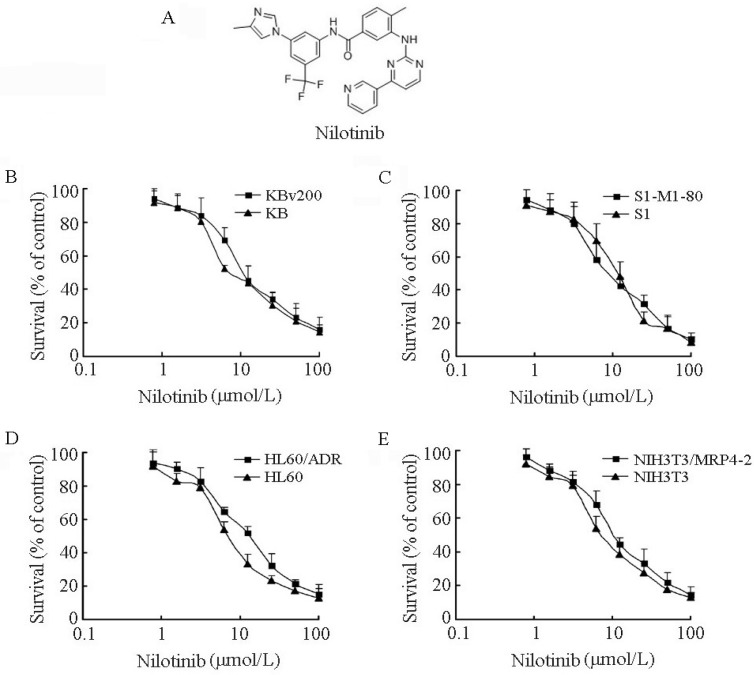
The structure and cytotoxic effects of nilotinib. (**A**) The structure of nilotinib. MTT cytotoxicity assay was assessed in pairs of parental and transporter- overexpressing cell lines: (**B**) KB and ABCB1-overexpressing KBv200 cells. (**C**) S1 and ABCG2-overexpressing S1-M1-80 cells. (**D**) HL60 and ABCC1-overexpressing HL60/ADR cells. (**E**) NIH3T3 and ABCC4-overexpressing NIH3T3/MRP4-2 cells. All the cells were exposed to full-range concentrations of nilotinib for 72 h. Each point represents the mean ± standard deviations (SDs) for three determinations. Each experiment was performed in three replicate wells.

The cytotoxicity of nilotinib in different cell lines was determined by the MTT assay. No significant difference in the cytotoxicity of nilotinib was observed between the parental sensitive and resistant cells. The IC_50_ values of nilotinib for KB, KBv200, S1, S1-M1-80, HL60, HL60/ADR, NIH3T3 and NIH3T3/MRP4 cells were 8.74 ± 0.76, 12.99 ±1.38, 10.74 ± 0.92, 9.59 ± 0.58, 8.49 ± 0.22, 13.59 ± 1.94, 8.85 ± 0.53, 13.36 ± 1.32 µmol/L, respectively (Figure 4). Based on the cytotoxicity curves, more than 85% of cells were viable when treated with nilotinib alone up to 2.0 µmol/L in all of the eight cell lines. Therefore, nilotinib at 0.5, 1.0 and 2.0 µmol/L was chosen for the MDR reversal study. As shown in Table 1, nilotinib produced a concentration-dependent decrease in the IC_50_ values of (*a*) doxorubicin and daunorubicin (substrates of ABCB1) in KBv200 cells; (*b*) mitoxantrone and topotecan (substrates of ABCG2) in S1-M1-80 cells. At the presence of 2.0 µmol/L nilotinib, the IC_50_ values of doxorubicin and daunorubicin in KBv200 cells reduced from 18.51 ± 0.337 to 0.876 ± 0.075 µmol/L and from 17.33 ± 0.044 to 1.223 ± 0.086 µmol/L respectively, representing a 21.1-and 14.2-fold drug sensitization. Similarly, 2.0 µmol/L nilotinib significantly decreased the IC_50_ of mitoxantrone and topotecan in S1-M1-80 cells from 23.60 ± 0.980 to 0.590 ± 0.052 µmol/L and from 25.43 ± 5.442 to 1.78 ± 0.372 µmol/L respectively, representing a 40.0- and 14.3-fold drug sensitization. In contrast, nilotinib did not alter the cytotoxicity of the antineoplastic drugs in the sensitive parental cells. Furthermore, nilotinib did not significantly alter the cytotoxicity of non-ABCB1 and non-ABCG2 substrate (cisplatin) in either MDR cells or their parental sensitive cells.

**Table 1:** Effect of nilotinib on reversing ABCB1-, ABCG2-, ABCC1- and ABCC4- mediated drug resistance.

Compounds	IC_50_ ± SD (μM) (Fold-Reversal)				
	KB		KBv200 (ABCB1)		
Doxorubicin	0.998 ± 0.013	(1.00)		18.51 ± 0337	(1.00)
+ 0.5 μM Nilotinib	0.874 ± 0.055	(1.14)		4.325 ± 0.047	(4.28) **
+ 1.0 μM Nilotinib	0.540 ± 0.014	(1.85) *		2.135 ± 0.065	(8.67) **
+ 2.0 μM Nilotinib	0.363 ± 0.006	(2.75) *		0.876 ± 0.075	(21.1) **
+ 10 μM Verapamil	0.629 ± 0.008	(1.57)		1.351 ± 0.036	(13.7) **
Daunorubicin	0.493 ± 0.42	(1.00)		17.33 ± 0.044	(1.00)
+ 0.5 μM Nilotinib	0.312 ± 0.020	(1.58)		5.252 ± 0.135	(3.30) **
+ 1.0 μM Nilotinib	0.298 ± 0.084	(1.65) *		3.365 ± 0.097	(5.15) **
+ 2.0 μM Nilotinib	0.215 ± 0.039	(2.29) *		1.223 ± 0.086	(14.2) **
+ 10 μM Verapamil	0.351 ± 0.068	(1.40)		0.873 ± 0.078	(19.9) **
Cisplatin	1.682 ± 0.073	(1.00)		2.632 ± 0.261	(1.00)
+ 2.0 μM Nilotinib	1.567 ± 0.142	(0.93)		2.346 ± 0.218	(0.89)
	S1			S1-M1-80 (ABCG2)	
Mitoxantrone	0.310 ± 0.031	(1.00)		23.60 ± 0.980	(1.00)
+ 0.5 μM Nilotinib	0.286 ± 0.074	(1.08)		5.626 ± 0.035	(4.19) **
+ 1.0 μM Nilotinib	0.245 ± 0.041	(1.26)		1.405 ± 0.039	(16.8) **
+ 2.0 μM Nilotinib	0.243 ± 0.036	(1.28)		0.590 ± 0.052	(40.0) **
+ 2.5 μM FTC	0.238 ± 0.013	(1.30)		0.289 ± 0.023	(81.7) **
Topotecan	0.365 ± 4.381	(1.00)		25.43 ± 5.442	(1.00)
+ 0.5 μM Nilotinib	0.297 ± 0.063	(1.23)		7.27 ± 0.141	(3.50) **
+ 1.0 μM Nilotinib	0.287 ± 0.034	(1.27)		4.19 ± 0.451	(6.07) **
+ 2.0 μM Nilotinib	0.208 ± 0.019	(1.75)		1.78 ± 0.372	(14.3) **
+ 2.5 μM FTC	0.245 ± 0.032	(1.49)		0.48 ± 0.012	(53.0) **
Cisplatin	14.788 ±1.678	(1.00)		16.428 ± 1.851	(1.00)
+ 2.0 μM Nilotinib	14.118±1.335	(0.95)		15.390 ± 1.356	(0.94)
	HL60			HL60/ADR (ABCC1)	
Doxorubicin	0.059 ± 0.009	(1.00)		1.191 ± 0.074	(1.00)
+ 0.5 μM Nilotinib	0.069 ± 0.002	(0.86)		1.009 ± 0.032	(1.18)
+ 1.0 μM Nilotinib	0.040 ± 0.006	(1.48)		0.775 ± 0.074	(1.54)
+ 2.0 μM Nilotinib	0.058 ± 0.016	(1.02)		0.862 ± 0.076	(1.38)
+ 50 μM MK571	0.039 ± 0.003	(1.51)		0.069 ± 0.007	(17.3) **
	NIH3T3			NIH3T3/MRP4 (ABCC4)	
6-Mercaptopurine	0.029 ± 0.002	(1.00)		0.32 ± 0.005	(1.00)
+ 0.5 μM Nilotinib	0.025 ± 0.004	(1.16)		0.30 ± 0.002	(1.06)
+ 1.0 μM Nilotinib	0.030 ± 0.001	(0.98)		0.31 ± 0.001	(1.03)
+ 2.0 μM Nilotinib	0.028 ± 0.003	(1.04)		0.24 ± 0.003	(1.33)

Cell survival was determined by MTT. Data are the mean ± standard deviations (SDs) of at least three independent experiments performed in triplicate. The fold reversal of MDR was calculated by dividing the IC_50_ value for cells with the anticancer drug in the absence of nilotinib by that obtained in the presence of nilotinib. * *p* < 0.05; ** *p* < 0.01.

In addition, nilotinib showed no reversal effect on ABCC1- or ABCC4-mediated drug resistance in HL60/ADR and NIH3T3/MRP4-2 cells. Taken together, though a slight synergistic effect was observed in KBv200 and KB cells when treated with nilotinib in combination with conventional chemotherapeutic agents, nilotinib significantly sensitized ABCB1- or ABCG2-overexpressing cells to antineoplastic drugs.

### 2.5. Nilotinib was Less Effective at Inhibiting Bcr-Abl Kinase in CD34^+^CD38^−^ Cells

**Figure 5:**
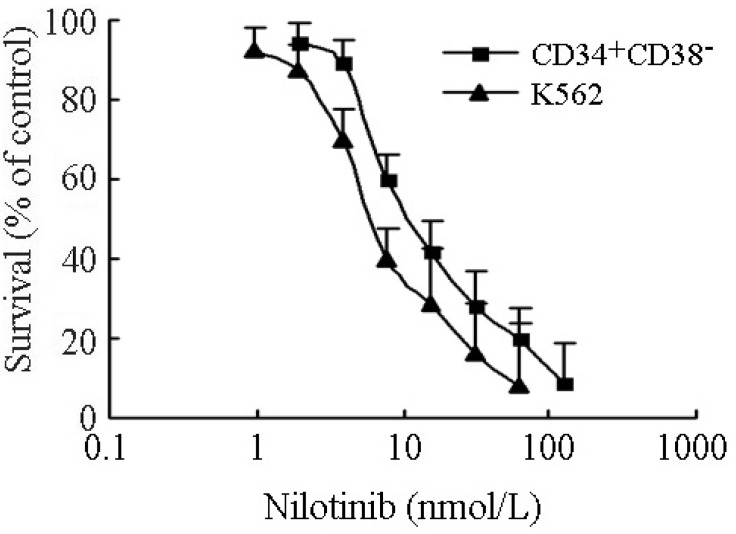
The cytotoxic effect of nilotinib on CD34^+^CD38^−^ and parental K562 cells. The MTT cytotoxicity assay was assessed in CD34^+^CD38^−^ leukemic subpopulation isolated from K562 cells and parental K562 cells. All the cells were exposed to full-range concentrations of nilotinib for 72 h. Each point represents the mean ± standard deviations (SDs) for three determinations. Each experiment was performed in three replicate wells.

**Figure 6:**
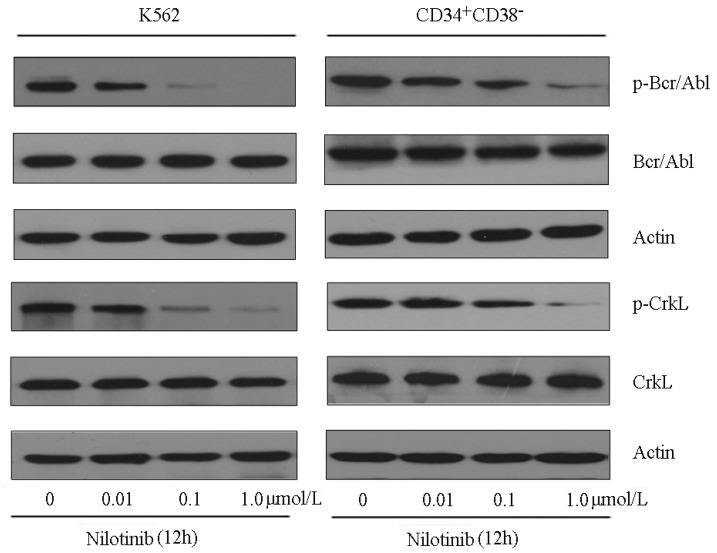
Effect of nilotinib on Bcr-Abl kinase activity in ABCB1- and ABCG2- overexpressing CD34^+^CD38^−^ cells. K562 parental cells and CD34^+^CD38^−^ subpopulation isolated from K562 cells were treated with nilotinib at 0.01, 0.1 and 1.0 μmol/L for 12 h. Equal amount of protein was loaded for western blot analysis as described in the Experimental section. The experiments were repeated at least three times independently, and a representative experiment is shown.

Results from cytotoxicity assays showed that CD34^+^CD38^−^ leukemic subpopulation isolated from K562 cells exhibited moderate resistance (2.41-fold) to nilotinib, relative to parental K562 cells. The IC_50_ values of nilotinib for CD34^+^CD38^−^and K562 cells were 17.40 ± 3.15 nmol/L and 7.21 ± 1.28 nmol/L, respectively (Figure 5). Since nilotinib targets the Bcr-Abl kinase in CML cells, we evaluated its ability to inhibit kinase activity in ABCB1- and ABCG2-overexpressing CD34^+^CD38^−^ cells. In K562 cells, nilotinib effectively inhibited the phosphorylation of Bcr-Abl and CrkL (a surrogate marker of Bcr-Abl activity) at a concentration of 0.1 µmol/L. However, in CD34^+^CD38^−^ cells, nilotinib failed to completely inhibit the phosphorylation of Bcr-Abl and CrkL even when cells were exposed to concentration up to 1.0 µmol/L (Figure 6). The level of total Bcr-Abl and CrkL remained unchanged in these cells after treated with nilotinib.

### 2.6. Discussion

Standard induction therapy is commonly used to produce complete remission and to prolong survival in patients with acute leukemia. However, many patients are either resistant to any initial treatment or acquire resistance to chemotherapy over time. In some previous published reports, combination therapy employing TKIs and classical chemotherapeutic drugs has led to improved anti-leukemic effects by inhibiting the functions of ABC transporters. Nilotinib is a second-generation inhibitor of the Bcr-Abl tyrosine kinase activity and has been approved for imatinib-resistant and -intolerant chronic myeloid leukemia patients in the chronic and accelerated phases of the disease [40,41]. The objective of this study was to examine the specific interaction of nilotinib with ABC transporters and its ability to eradicate leukemic stem cells.

Leukemic stem cells comprise a side population (SP) of haematopoietic cells that are able to survive chemotherapy and sustain the disease [42,43,44]. Incomplete chemotherapeutic eradication of leukemia-initiating CD34^+^CD38^−^ cells is associated with an increased risk of relapse and resistance in acute leukemia patients. The relative high expression level of ABC transporters in CD34^+^CD38^−^ cells suggested that these proteins might be potent targets for stem cell eradication in leukemia patients [20,45,46]. To test this hypothesis, we assessed the effect of nilotinib on modulating chemosensitivity in leukemic CD34^+^CD38^−^ cells. As shown in Figure 1, CD34^+^CD38^−^ cell subsets were more resistant to doxorubicin and mitoxantrone than CD34^+^CD38^−^ cells. The inhibition ratio of doxorubicin and mitoxantrone at 1.0 μmol/L in CD34^+^CD38^−^ cells were 26.8% and 26.7%, while slight cytotoxic effect was observed in CD34^+^CD38^−^ cells. Nilotinib at 2.0 μmol/L sensitized CD34^+^CD38^−^ cells to doxorubicin and mitoxantrone by 10.5- and 9.3-fold, respectively. Furthermore, nilotinib significantly increased doxorubicin-induced apoptosis in leukemic CD34^+^CD38^−^ cells.

Co-expression of ABCB1 and ABCG2 is associated with lower complete response (CR) rate and with worse event-free survival and overall survival [8,47]. In our study, the high expression of four ABC genes including ABCB1, ABCG2, ABCC1 and ABCC4 was detected in patients with newly diagnosed acute leukemia. As shown in Figure 2, co-expression of the four genes was detected in three patient samples. Importantly, capable of completely blocking ABCB1 and ABCG2 as confirmed in the drug-resistant cell models, it is noteworthy that in the *ex vivo* model of ABCB1- and ABCG2 overexpressing primary leukemic blast cells, nilotinib significantly enhanced the cytotoxicity of doxorubicin (Figure 3). 

In accordance with previous reports that nilotinib was a potent inhibitor of ABCB1 and ABCG2 [48,49,50,51,52]. Our results showed that nilotinib had potent reversing activity in ABCB1- and ABCG2-mediated MDR *in vitro*. However, no reversal effect was observed in ABCC1-overexpressing HL60/ADR cells and ABCC4-overexpressing NIH3T3/ABCC4-2 cells. Nilotinib at 2.0 μmol/L significantly increased the sensitivity of ABCB1-overexpressing KBv200 cells to doxorubicin and daunorubicin by 21.1- and 14.2-fold, respectively. In ABCG2-overexpressing S1-M1-80 cells, 2.0 μmol/L nilotinib produced a 40.0-fold mitoxantrone sensitization and a 14.3-fold topotecan sensitization. Additionally, nilotinib did not alter cellular sensitivity to cisplatin (a non-substrate of ABCB1 and ABCG2) and showed no reversal effect in the parental drug-sensitive cells, indicating that the sensitizing activity of nilotinib in MDR cells was attributed to its specific effect on ABCB1 and ABCG2 (Table 1). 

The data above strongly indicated that nilotinib could target CD34^+^CD38^−^ and ABC transporter overexpressing leukemia cells, and effectively enhanced the cytotoxicity of conventional anticancer drugs. Further studies showed that nilotinib produced a significant concentration-dependent increase in accumulation of Rho 123 in ABCB1- and/or ABCG2-overexpressing primary leukemic blasts (Figure 3D). We also demonstrated that ABCB1- and ABCG2-overexpression mediated moderate resistance to nilotinib in CD34^+^CD38^−^ cells (Figure 5). Besides, nilotinib was less effective at inhibiting the phosphorylation of Bcr-Abl and CrkL in CD34^+^CD38^−^ cells, compared to that in parental control K562 cells (Figure 6). These observations suggested that overexpression of ABC transporters could contribute to nilotinib resistance in CD34^+^CD38^−^ cells.

In conclusion, nilotinib could effectively enhance the chemosensitivity of classical chemotherapeutic drugs in CD34^+^CD38^−^ stem cells and ABC transporter-overexpressing leukemia cells *via* directly inhibiting the drug transport function of ABCB1 and ABCG2. Our results suggest that nilotinib can be used in conjunction with conventional ABCB1 and ABCG2 substrate anticancer drugs to combat the problem of multidrug resistance in the clinic. Moreover, overexpression of ABC transporters could cause inherent resistance to nilotinib in leukemia stem cells, suggesting that the interaction of nilotinib with ABC transporters may be an important factor in the treatment of leukemia patients

## 3. Experimental

### 3.1. Chemicals and Reagents

Nilotinib was purchased from Selleck Chemicals (Houston, TX, USA). Dulbecco’s modified Eagle’s medium (DMEM) and RPMI 1640 were products of Gibco BRL (Gaithersburg, MD, USA). 3-(4,5-Dimethylthiazol-yl)-2,5-diphenyllapatinibrazolium bromide (MTT), daunorubicin, doxorubicin (Dox), topotecan, mitoxantrone, 6-mercaptopurine, rhodamine 123 (Rho 123), verapamil (VRP), cisplatin, MK571 and fumitremorgin C (FTC) were products of Sigma Chemical Co (St. Louis, MO, USA). Monoclonal antibodies against ABCB1 and ABCG2 were purchased from Santa Cruz Biotechnology, Inc. (Delaware Ave, CA, USA). Antibody against glyceraldehyd-3-phosphate dehydrogenase (GAPDH) was purchased from Kangcheng Co. (Shanghai, China). VRP, FTC and MK571 (inhibitor for ABCB1, ABCG2 and ABCC1, respectively) were used in place of nilotinib as positive control to confirm the mechanism of drug resistance in the MDR cell line models.

### 3.2. Cell Lines and Culture Conditions

The following cell lines were cultured in DMEM or RPMI 1640 containing 10% fetal bovine serum, 100 units/mL penicillin, 100 units/mL streptomycin, at 37 °C in the presence of 5% CO_2_: the human colon carcinoma cell line S1 and its mitoxantrone-selected derivative ABCG2-overexpressing S1-M1-80 cell line were kindly provided by S.E. Bates (National Cancer Institute, National Institutes of Health [NIH], Bethesda, MD, USA) [53]; the human oral epidermoid carcinoma cell line KB and its vincristine-selected derivative ABCB1-overexpressing cell line KBv200 were a gift from Xu-Yi Liu (Cancer Hospital of Beijing, Beijing, China); the human leukemia cell line HL60 and its Dox-selected derivative ABCC1-overexpressing cell line HL60/ADR were purchased from the Institute of Hematology & Blood Diseases Hospital, Chinese Academy of Medical Sciences & Peking Union Medical College (Tianjin, China); the murine fibroblasts cell line NIH3T3 and the ABCC4-transfected ABCC4 stable expressing NIH3T3/MRP4-2 cells were kindly provided by Z-S Chen (St John’s University, Queens, NY, USA) [54]. 

### 3.3. Patient Samples

Bone marrow samples from diagnosed AML or ALL patients according to the French-American-British (FAB) classification were collected with informed consent, and this study was approved by the Ethics Review Committee at Sun Yat-Sen University. Leukemic blasts were isolated using Ficoll-Hypaque density gradient by centrifugation and cultured in RPMI 1640 medium containing 10% fetal bovine serum, 100 units/mL penicillin, 100 units/mL streptomycin, at 37 °C in the presence of 5% CO_2_.

### 3.4. Cell Proliferation Assay

The MTT assay was used for the assessment of cell proliferation with minor modifications as described previously [38]. After 68 h of incubation with tested drugs, MTT (5 mg/mL, 20 μL/well) was added into the cells for 4 h (37 °C). Afterwards, the medium was discarded, and 200 μL of dimethylsulfoxide (DMSO) was added to dissolve the formazan product from metabolism of MTT. Optical density was measured at 540 nm with background subtraction at 670 nm by use of the Model 550 Microplate Reader (Bio-Rad, Hercules, CA, USA). The concentration required to inhibit cell growth by 50% (IC_50_) was calculated from survival curves by use of the Bliss method. For reversal experiments, nilotinib was added to the medium with full range concentrations of: (1) doxorubicin, daunorubicin and cisplatin in KB and KBv200; (2) topotecan, mitoxantrone and cisplatin in S1 and S1-M1-80; (3) doxorubicin in HL60 and HL60/ADR; (4) 6-mercaptopurine (6-MP) in NIH3T3 and NIH3T3/MRP4-2 cells. Fold of resistance was calculated by dividing the IC_50_ for the MDR cells by that for the parental sensitive cells. The degree of reversal of MDR (fold-reversal) was calculated by dividing the IC_50_ for cells with the anticancer drug in the absence of nilotinib by that obtained in the presence of nilotinib.

### 3.5. CD34^+^CD38^−^ Cell Analysis and Sorting

BM mononuclear cells were isolated using Ficoll-Hypaque density gradient separation. Then cells (1 × 10^6^/tube) were incubated with combinations of PE-labeled CD34 monoclonal antibody (mAb) and APC-conjugated CD38^−^ mAb for 15 min, cells were then washed and resuspended in PBS. CD34^+^CD38^−^ or primitive progenitor cells were isolated by flow cytometric sorting. 

### 3.6. Apoptosis Assay

Assessment of apoptosis rates was done by annexin V/propidium iodide (PI) staining using the annexin V-FITC Kit. Cells were seeded onto a six-well plate at a density of about 2.0 × 10^5^ cells/well. After treatment with different concentrations of nilotinib in the presence of 1.0 μmol/L doxorubicin or mitoxantrone for 48 h, both floating and attached cells were collected and washed with ice-cold PBS twice. Cells were resuspended in 100 μL of 1× binding buffer, and the Alexa Fluoro 488 annexin V (5 μL) and PI (1 μL) were added before incubation at room temperature for 15 min. Then cells were resuspended in 400 μL 1× binding buffer, mixed gently and analyzed via fluorescence-activated cell sorting (FACS). 

### 3.7. Rhodamine 123 Accumulation

The effect of nilotinib on the fluorescent dye transport capacities of ABCB1 and ABCG2 was followed as published previously. Briefly, the cells were treated with nilotinib of various concentrations or vehicle at 37 °C for 3 h. Subsequently, 5 μmol/L rhodamine 123 was added and the incubation was continued for an additional 0.5 h. Cellular fluorescence was analyzed with flow cytometric analysis (Cytomics FC500, Beckman Coulter, Fullerton, CA, USA). ABCB1 inhibitor verapamil (10 μmol/L) or ABCG2 inhibitor FTC (2 μmol/L) was used as positive control. 

### 3.8. Reverse Transcription-and Quantitative Real-Time-Polymerase Chain Reaction

Total RNA were extracted by use of a Trizol Reagent RNA extraction kit according to the manufacturer’s instructions (Molecular Research Center, Cincinnati, OH, USA). The first strand cDNA was synthesized by Oligo dT primers with reverse transcriptase (Promega Corp., Madison, WI, USA). Oligonucleotide primers for ABC transporter genes and glyceraldehyde-3-phosphate dehydrogenase (GAPDH) were synthesized commercially (Invitrogen Co., Guangzhou, China). Using the GeneAmp PCR system 9700 (PE Applied Biosystems, Foster City, CA, USA), reactions were carried out at 94 °C for 2 min for initial denaturation, and then at 94 °C for 30 s, 58 °C for 30 s, and 72 °C for 1 min. After 35 cycles of amplification, additional extensions were carried out at 72 °C for 10 min. Products were resolved and examined by 1.5% agarose gel electrophoresis. 

Real-time PCR was performed with Real-time PCR Master Mix containing SYBR GREEN I and hotstart Taq DNA poly-merase. The thermal cycling was as follows: 10 min at 95 °C, followed by 40 cycles of 10 s at 94 °C, 30 s at 55–60 °C and 30 s at 72 °C. To verify the specific amplification, melting curve analysis was performed (55–95 °C, 0.5 °C/s). Relative quantification was performed by the ΔΔCT method. The sequence information of primers used is available in Table 2. The expression of four MDR genes (ABCB1, ABCG2, ABCC1 and ABCC4) was analyzed and GAPDH was used as internal control.

**Table 2:** The primers and probes sequence for quantitative real-time PCR.

Name	Sequence
ABCB1-F	5'-CTGGACAAGCACTGAAAGATAAGA-3'
ABCB1-R	5'-CAACGGTTCGGAAGTTTTCT-3'
ABCB1-P	5'-FAM-TCTGGGAAGATCGCTACTGAAGCA-TAMRA-3'
ABCG2-F	5'-CAGTACTTCAGCATTCCACGAT-3'
ABCG2-R	5'-GGCAGAAGTTTTGTCCCAAA-3'
ABCG2-P	5'-FAM-CATTATGCTGCAAAGCCGTAAATCCA-TAMRA-3'
ABCC1-F	5'-CATGGTGCCCGTCAATG-3'
ABCC1-R	5'-CGATTGTCTTTGCTCTTCATGT-3'
ABCC1-P	5'-FAM-TGGCGATGAAGACCAAGACGTATCAGGT-TAMRA-3'
ABCC4-F	5'-TGCCATCTGTGCCATGTTT-3'
ABCC4-R	5'-CCAGAGTTTTTGCCAGAATCA-3'
ABCC4-P	5'-FAM-TCATCATCGTTGCCTTTGGGTCC-TAMRA-3'
GAPDH-F	5'-ATGCCCCCATGTTTGTGATG-3′
GAPDH-R	5'-TCCTCAGTGTAGCCCAAGATGC-3′
GAPDH-P	5'-FAM-CAAGCT TCC CGT TCT CAGCC-TAMRA-3

### 3.9. Detection of Cell Surface Expression of ABCG2 and ABCB1 by Flow Cytometer

For ABCG2 expression analysis, APC-conjugated anti-human Bcrp1/ABCG2 (R&D Systems, Minneapolis, MN, USA) reagent was mixed with 25 μL of Fc-blocked cells (1 × 10^6^ cells). After incubating for 45 min at 4 °C, cells were washed twice with PBS buffer (supplemented with 0.5% BSA) and resuspended in 400 μL PBS buffer for flow cytometric analysis. Isotype control samples were treated in an identical manner with allophycocyanin (APC)-labeled mouse immunoglobin G2b (IgG2b) antibody. For ABCB1 flow cytometric analysis, 1 × 10^6^ cells (100 μL) were incubated at 4 °C for 30 min with 10 μL of CD243-PE conjugated antibody (Beckman Coulter), cells were then washed and resuspended in PBS. Isotype control samples were treated with mouse IgG2a antibody in parallel. Tests and controls were analyzed with a flow cytometer.

### 3.10. Western Blot Analysis

To determine the effect of nilotinib on the phosphorylation of Bcr-Abl protein and its signal transduction, CD34^+^CD38^−^ and K562 cells were incubated with 0.01, 0.1, and 1.0 μmol/L nilotinib for 12 h. Then, whole cell lysates were harvested and washed twice with ice-cold PBS. Cell extracts were collected in cell lysis buffer (PBS containing 1% Nonidet P-40, 0.5% sodium deoxycholate, 0.1% SDS, 100 mg/mL PMSF, 10 mg/mL aprotinin, 10 mg/mL leupeptin). Equal amounts of protein were resolved by SDS-polyacrylamide gel electrophoresis and transferred to nitrocellulose membranes. After blocking in TBST (10 mmol/L Tris-HCl, 150 mmol/L NaCl and 0.1% Tween 20, pH 8.0) with 5% non-fat milk for 2 h at room temperature, the membranes were incubated with appropriately diluted primary antibodies overnight at 4 °C. The membranes were then washed three times with TBST and incubated with HRP-conjugated secondary antibody at 1:5000 dilutions for 2 h at room temperature. After three washes with TBST, the protein-antibody complexes were visualized by the enhanced Phototope TM-HRP Detection Kit (Cell Signaling, Rockford, IL, USA) and exposed to Kodak medical X-ray processor (Carestream Health, Atlanta, GA, USA). GAPDH was used as a loading control. 

### 3.11. Statistical Analysis

Three to five independent experiments were performed and the results were depicted as mean value ± standard deviations (SDs). Statistical significant differences of the data were calculated using the Student *t* test with significance levels of *p* < 0.05 and *p* < 0.01.

## 4. Conclusions

Nilotinib particularly targets CD34^+^CD38^−^ stem cells and MDR leukemia cells, and effectively enhances the efficacy of chemotherapeutic drugs by blocking the efflux function of ABC transporters. 

## References

[1] Rees D.C., Johnson E., Lewinson O. (2009). ABC transporters: The power to change. Nat. Rev. Mol. Cell Biol..

[2] Sarkadi B., Homolya L., Szakacs G., Varadi A. (2006). Human multidrug resistance ABCB and ABCG transporters: Participation in a chemoimmunity defense system. Physiol. Rev..

[3] Szakacs G., Varadi A., Ozvegy-Laczka C., Sarkadi B. (2008). The role of ABC transporters in drug absorption, distribution, metabolism, excretion and toxicity (ADME-Tox). Drug Discov. Today.

[4] Gottesman M.M., Fojo T., Bates S.E. (2002). Multidrug resistance in cancer: Role of ATP-dependent transporters. Nat. Rev. Cancer.

[5] Damiani D., Tiribelli M., Raspadori D., Michelutti A., Gozzetti A., Calistri E., Candoni A., Chiarvesio A., Lenoci M., Russo D. (2007). The role of MDR-related proteins in the prognosis of adult acute myeloid leukaemia (AML) with normal karyotype. Hematol. Oncol..

[6] Huh H.J., Park C.J., Jang S., Seo E.J., Chi H.S., Lee J.H., Lee K.H., Seo J.J., Moon H.N., Ghim T. (2006). Prognostic significance of multidrug resistance gene 1 (MDR1), multidrug resistance-related protein (MRP) and lung resistance protein (LRP) mRNA expression in acute leukemia. J. Korean Med. Sci..

[7] Schaich M., Soucek S., Thiede C., Ehninger G., Illmer T. (2005). MDR1 and MRP1 gene expression are independent predictors for treatment outcome in adult acute myeloid leukaemia. Br. J. Haematol..

[8] Benderra Z., Faussat A.M., Sayada L., Perrot J.Y., Chaoui D., Marie J.P., Legrand O. (2004). Breast cancer resistance protein and P-glycoprotein in 149 adult acute myeloid leukemias. Clin. Cancer Res..

[9] Steinbach D., Gillet J.P., Sauerbrey A., Gruhn B., Dawczynski K., Bertholet V., de Longueville F., Zintl F., Remacle J., Efferth T. (2006). ABCA3 as a possible cause of drug resistance in childhood acute myeloid leukemia. Clin. Cancer Res..

[10] Van der Kolk D.M., Vellenga E., Scheffer G.L., Muller M., Bates S.E., Scheper R.J., de Vries E.G. (2002). Expression and activity of breast cancer resistance protein (BCRP) in de novo and relapsed acute myeloid leukemia. Blood.

[11] Mahjoubi F., Golalipour M., Ghavamzadeh A., Alimoghaddam K. (2008). Expression of MRP1 gene in acute leukemia. Sao Paulo Med. J..

[12] Plasschaert S.L., de Bont E.S., Boezen M., vander Kolk D.M., Daenen S.M., Faber K.N., Kamps W.A., de Vries E.G., Vellenga E. (2005). Expression of multidrug resistance-associated proteins predicts prognosis in childhood and adult acute lymphoblastic leukemia. Clin. Cancer Res..

[13] Tafuri A., Gregorj C., Petrucci M.T., Ricciardi M.R., Mancini M., Cimino G., Mecucci C., Tedeschi A., Fioritoni G., Ferrara F. (2002). MDR1 protein expression is an independent predictor of complete remission in newly diagnosed adult acute lymphoblastic leukemia. Blood.

[14] Pietras A. (2011). Cancer stem cells in tumor heterogeneity. Adv. Cancer Res..

[15] Clevers H. (2011). The cancer stem cell: Premises, promises and challenges. Nat. Med..

[16] Buss E.C., Ho A.D. (2011). Leukemia stem cells. Int. J. Cancer.

[17] Cheng L., Alexander R., Zhang S., Pan C.X., MacLennan G.T., Lopez-Beltran A., Montironi R. (2011). The clinical and therapeutic implications of cancer stem cell biology. Expert Rev. Anticancer Ther..

[18] Lapidot T., Sirard C., Vormoor J., Murdoch B., Hoang T., Caceres-Cortes J., Minden M., Paterson B., Caligiuri M.A., Dick J.E. (1994). A cell initiating human acute myeloid leukaemia after transplantation into SCID mice. Nature.

[19] Bonnet D., Dick J.E. (1997). Human acute myeloid leukemia is organized as a hierarchy that originates from a primitive hematopoietic cell. Nat. Med..

[20] Dean M., Fojo T., Bates S. (2005). Tumour stem cells and drug resistance. Nat. Rev. Cancer.

[21] Smeets M., Raymakers R., Vierwinden G., Pennings A., van de Locht L., Wessels H., Boezeman J., de Witte T. (1997). A low but functionally significant MDR1 expression protects primitive haemopoietic progenitor cells from anthracycline toxicity. Br. J. Haematol..

[22] Scharenberg C.W., Harkey M.A., Torok-Storb B. (2002). The ABCG2 transporter is an efficient Hoechst 33342 efflux pump and is preferentially expressed by immature human hematopoietic progenitors. Blood.

[23] Zhou S., Schuetz J.D., Bunting K.D., Colapietro A.M., Sampath J., Morris J.J., Lagutina I., Grosveld G.C., Osawa M., Nakauchi H. (2001). The ABC transporter Bcrp1/ABCG2 is expressed in a wide variety of stem cells and is a molecular determinant of the side-population phenotype. Nat. Med..

[24] Kim M., Turnquist H., Jackson J., Sgagias M., Yan Y., Gong M., Dean M., Sharp J.G., Cowan K. (2002). The multidrug resistance transporter ABCG2 (breast cancer resistance protein 1) effluxes Hoechst 33342 and is overexpressed in hematopoietic stem cells. Clin. Cancer Res..

[25] De Grouw E.P., Raaijmakers M.H., Boezeman J.B., van der Reijden B.A., van de Locht L.T., de Witte T.J., Jansen J.H., Raymakers R.A. (2006). Preferential expression of a high number of ATP binding cassette transporters in both normal and leukemic CD34^+^CD38^−^ cells. Leukemia.

[26] Raaijmakers M.H. (2007). ATP-binding-cassette transporters in hematopoietic stem cells and their utility as therapeutical targets in acute and chronic myeloid leukemia. Leukemia.

[27] Good J.R., Kuspa A. (2000). Evidence that a cell-type-specific efflux pump regulates cell differentiation in Dictyostelium. Dev. Biol..

[28] Tsuruo T., Iida H., Tsukagoshi S., Sakurai Y. (1981). Overcoming of vincristine resistance in P388 leukemia *in vivo* and *in vitro* through enhanced cytotoxicity of vincristine and vinblastine by verapamil. Cancer Res..

[29] Shaffer B.C., Gillet J.P., Patel C., Baer M.R., Bates S.E., Gottesman M.M. (2012). Drug resistance: Still a daunting challenge to the successful treatment of AML. Drug Resist. Updat..

[30] Dai C.L., Tiwari A.K., Wu C.P., Su X.D., Wang S.R., Liu D.G., Ashby C.R., Huang Y., Robey R.W., Liang Y.J. (2008). Lapatinib (Tykerb, GW572016) reverses multidrug resistance in cancer cells by inhibiting the activity of ATP-binding cassette subfamily B member 1 and G member 2. Cancer Res..

[31] Kuang Y.H., Shen T., Chen X., Sodani K., Hopper-Borge E., Tiwari A.K., Lee J.W., Fu L.W., Chen Z.S. (2010). Lapatinib and erlotinib are potent reversal agents for MRP7 (ABCC10)-mediated multidrug resistance. Biochem. Pharmacol..

[32] Kitazaki T., Oka M., Nakamura Y., Tsurutani J., Doi S., Yasunaga M., Takemura M., Yabuuchi H., Soda H., Kohno S. (2005). Gefitinib, an EGFR tyrosine kinase inhibitor, directly inhibits the function of P-glycoprotein in multidrug resistant cancer cells. Lung Cancer.

[33] Nakamura Y., Oka M., Soda H., Shiozawa K., Yoshikawa M., Itoh A., Ikegami Y., Tsurutani J., Nakatomi K., Kitazaki T. (2005). Gefitinib (“Iressa”, ZD1839), an epidermal growth factor receptor tyrosine kinase inhibitor, reverses breast cancer resistance protein/ABCG2-mediated drug resistance. Cancer Res..

[34] Shi Z., Peng X.X., Kim I.W., Shukla S., Si Q.S., Robey R.W., Bates S.E., Shen T., Ashby C.R., Fu L.W. (2007). Erlotinib (Tarceva, OSI-774) antagonizes ATP-binding cassette subfamily B member 1 and ATP-binding cassette subfamily G member 2-mediated drug resistance. Cancer Res..

[35] Tao L.Y., Liang Y.J., Wang F., Chen L.M., Yan Y.Y., Dai C.L., Fu L.W. (2009). Cediranib (recentin, AZD2171) reverses ABCB1- and ABCC1-mediated multidrug resistance by inhibition of their transport function. Cancer Chemother. Pharmacol..

[36] Zheng L.S., Wang F., Li Y.H., Zhang X., Chen L.M., Liang Y.J., Dai C.L., Yan Y.Y., Tao L.Y., Mi Y.J. (2009). Vandetanib (Zactima, ZD6474) antagonizes ABCC1- and ABCG2-mediated multidrug resistance by inhibition of their transport function. PLoS One.

[37] Dai C.L., Liang Y.J., Wang Y.S., Tiwari A.K., Yan Y.Y., Wang F., Chen Z.S., Tong X.Z., Fu L.W. (2009). Sensitization of ABCG2-overexpressing cells to conventional chemotherapeutic agent by sunitinib was associated with inhibiting the function of ABCG2. Cancer Lett..

[38] Wang F., Mi Y.J., Chen X.G., Wu X.P., Liu Z., Chen S.P., Liang Y.J., Cheng C., To K.K., Fu L.W. (2012). Axitinib targeted cancer stemlike cells to enhance efficacy of chemotherapeutic drugs via inhibiting the drug transport function of ABCG2. Mol. Med..

[39] Tong X.Z., Wang F., Liang S., Zhang X., He J.H., Chen X.G., Liang Y.J., Mi Y.J., To K.K., Fu L.W. (2012). Apatinib (YN968D1) enhances the efficacy of conventional chemotherapeutical drugs in side population cells and ABCB1-overexpressing leukemia cells. Biochem. Pharmacol..

[40] Kantarjian H., Giles F., Wunderle L., Bhalla K., O’Brien S., Wassmann B., Tanaka C., Manley P., Rae P., Mietlowski W. (2006). Nilotinib in imatinib-resistant CML and Philadelphia chromosome-positive ALL. N. Engl. J. Med..

[41] Kantarjian H.M., Giles F., Gattermann N., Bhalla K., Alimena G., Palandri F., Ossenkoppele G.J., Nicolini F.E., O’Brien S.G., Litzow M. (2007). Nilotinib (formerly AMN107), a highly selective BCR-ABL tyrosine kinase inhibitor, is effective in patients with Philadelphia chromosome-positive chronic myelogenous leukemia in chronic phase following imatinib resistance and intolerance. Blood.

[42] Udomsakdi C., Eaves C.J., Sutherland H.J., Lansdorp P.M. (1991). Separation of functionally distinct subpopulations of primitive human hematopoietic cells using rhodamine-123. Exp. Hematol..

[43] Uchida N., Combs J., Chen S., Zanjani E., Hoffman R., Tsukamoto A. (1996). Primitive human hematopoietic cells displaying differential efflux of the rhodamine 123 dye have distinct biological activities. Blood.

[44] McKenzie J.L., Takenaka K., Gan O.I., Doedens M., Dick J.E. (2007). Low rhodamine 123 retention identifies long-term human hematopoietic stem cells within the Lin-CD34^+^CD38^−^ population. Blood.

[45] Engler J.R., Frede A., Saunders V.A., Zannettino A.C., Hughes T.P., White D.L. (2010). Chronic myeloid leukemia CD34^+^ cells have reduced uptake of imatinib due to low OCT-1 activity. Leukemia.

[46] Jiang X., Zhao Y., Smith C., Gasparetto M., Turhan A., Eaves A., Eaves C. (2007). Chronic myeloid leukemia stem cells possess multiple unique features of resistance to BCR-ABL targeted therapies. Leukemia.

[47] Damiani D., Tiribelli M., Calistri E., Geromin A., Chiarvesio A., Michelutti A., Cavallin M., Fanin R. (2006). The prognostic value of P-glycoprotein (ABCB) and breast cancer resistance protein (ABCG2) in adults with de novo acute myeloid leukemia with normal karyotype. Haematologica.

[48] Tiwari A.K., Sodani K., Wang S.R., Kuang Y.H., Ashby C.R., Chen X., Chen Z.S. (2009). Nilotinib (AMN107, Tasigna) reverses multidrug resistance by inhibiting the activity of the ABCB1/Pgp and ABCG2/BCRP/MXR transporters. Biochem. Pharmacol..

[49] Dohse M., Scharenberg C., Shukla S., Robey R.W., Volkmann T., Deeken J.F., Brendel C., Ambudkar S.V., Neubauer A., Bates S.E. (2010). Comparison of ATP-binding cassette transporter interactions with the tyrosine kinase inhibitors imatinib, nilotinib, and dasatinib. Drug Metab. Dispos..

[50] Brendel C., Scharenberg C., Dohse M., Robey R.W., Bates S.E., Shukla S., Ambudkar S.V., Wang Y., Wennemuth G., Burchert A. (2007). Imatinib mesylate and nilotinib (AMN107) exhibit high-affinity interaction with ABCG2 on primitive hematopoietic stem cells. Leukemia.

[51] Shukla S., Skoumbourdis A.P., Walsh M.J., Hartz A.M., Fung K.L., Wu C.P., Gottesman M.M., Bauer B., Thomas C.J., Ambudkar S.V. (2011). Synthesis and characterization of a BODIPY conjugate of the BCR-ABL kinase inhibitor Tasigna (nilotinib): Evidence for transport of Tasigna and its fluorescent derivative by ABC drug transporters. Mol. Pharm..

[52] Hegedus C., Ozvegy-Laczka C., Apati A., Magocsi M., Nemet K., Orfi L., Keri G., Katona M., Takats Z., Varadi A. (2009). Interaction of nilotinib, dasatinib and bosutinib with ABCB1 and ABCG2: Implications for altered anti-cancer effects and pharmacological properties. Br. J. Pharmacol..

[53] Litman T., Brangi M., Hudson E., Fetsch P., Abati A., Ross D.D., Miyake K., Resau J.H., Bates S.E. (2000). The multidrug-resistant phenotype associated with overexpression of the new ABC half-transporter, MXR (ABCG2). J. Cell Sci..

[54] Lee K., Klein-Szanto A.J., Kruh G.D. (2000). Analysis of the MRP4 drug resistance profile in transfected NIH3T3 cells. J. Natl. Cancer Inst..

